# *MYB* deregulation from a *EWSR1*-*MYB* fusion at leukemic evolution of a *JAK2*^V617F^ positive primary myelofibrosis

**DOI:** 10.1186/s13039-016-0277-1

**Published:** 2016-09-01

**Authors:** Tiziana Pierini, Danika Di Giacomo, Valentina Pierini, Paolo Gorello, Gianluca Barba, Anair Graciela Lema Fernandez, Fabrizia Pellanera, Tamara Iannotti, Franca Falzetti, Roberta La Starza, Cristina Mecucci

**Affiliations:** Hematology and Bone Marrow Transplantation Unit, University of Perugia, C.R.E.O., Piazzale Menghini n.9, 06132 Perugia, Italy

**Keywords:** PMF, Leukemic transformation, *MYB*

## Abstract

**Background:**

Although Philadelphia-negative myeloproliferative neoplasms (Ph-MPN) are usually not aggressive, the type and the number of molecular lesions impact greatly on leukemic transformation. Indeed, the molecular background underlying progression is still largely unexplored even though *ASXL1*, *IDH1/2, SRSF2*, and *TP53* mutations, together with adverse karyotypic changes, place the patient at high risk of leukemic transformation.

**Case presentation:**

Our patient, a 64-year old man with a diagnosis of *JAK2*^V617F^ primary myelofibrosis (PMF) had an unusually rapid leukemic transformation. Genomic profiling showed that *TET2* and *SRSF2* mutations were also present. At leukemic transformation, the patient developed a complex chromosome rearrangement producing a *EWSR1*-*MYB* fusion. Remarkably, the expression of *MYB* and of its target *BCL2* was, respectively, ≥4.7 and ≥2.8 fold higher at leukemic transformation than after chemotherapy, when the patient obtained the hematological remission. At this time point, the *EWSR1*-*MYB* fusion disappeared while *JAK2*^V617F^, *TET2,* and *SRSF2* mutations, as well as PMF morphological features persisted*.*

**Conclusions:**

Rapid leukemic transformation of *JAK2*^V617F^ PMF was closely linked to a previously undescribed putative EWSR1-MYB transcription factor which was detected only at disease evolution. We hypothesize that the EWSR1-MYB contributed to leukemia transformation through at least two mechanisms: 1) it sustained *MYB* expression, and consequently deregulated its target *BCL2*, a putative onco-suppressor gene*;* and 2) ectopic *EWSR1-MYB* expression probably fulfilled its own oncogenic potential as demonstrated for other *MYB*-fusions. As our study confirmed that *MYB* is recurrently involved in chronic as well as leukemic transformation of PMF, it appears to be a valid molecular marker for tailored treatments.

**Electronic supplementary material:**

The online version of this article (doi:10.1186/s13039-016-0277-1) contains supplementary material, which is available to authorized users.

## Background

Ph-MPN are usually not aggressive malignancies. Evolution into Acute Myeloid Leukemia (AML) occurs in ~2 % of Polycythemia Vera (PV), 1 % of Essential Thrombocytopenia (ET), and 10–20 % of PMF, considering the first decade of disease. Although, transformation rates increase with genotoxic therapy, leukemic transformation is part of the natural history of these disorders as AML also occurs in treatment-naive patients [1, 2].

Genomic profiling of Ph-MPN is useful for understanding the pathobiology of leukemic transformation and improving prognostic stratification of patients and treatment. During leukemic transformation very few new mutations are acquired and the majority of somatic mutations are already present in the chronic phase. Two or more mutations affecting *ASXL1, IDH1/2, SRSF2*, and *TP53*, are considered high-risk events. Besides mutations, unfavourable (chromosome 3, 5 or 7 rearrangements), and very unfavourable (chromosome 17 abnormalities) karyotypic changes impact upon prognosis [3, 4]. In any case, the molecular mechanisms underlying the progression from Ph-MPN to AML are not yet completely understood [5–9].

Longitudinal genomic investigation into a unique case of *JAK2*^V617F^ positive PMF with rapid leukemic evolution, detected a leukemia-specific complex rearrangement involving chromosomes 6, 9 and 22 which produced an aberrant EWSR1-MYB transcription factor. *EWSR1*-*MYB* ectopic expression, as well as high expression of *MYB* and its target *BCL2,* likely contributed to the leukemia phenotype. It is worth mentioning that, high level of *MYB* expression blocks differentiation and confers self-renewal properties to leukemic cells, whereas the expression of the anti-apoptotic BCL2 protein inhibits leukemic cell death [10–12].

## Case presentation

**Fig. 1:**
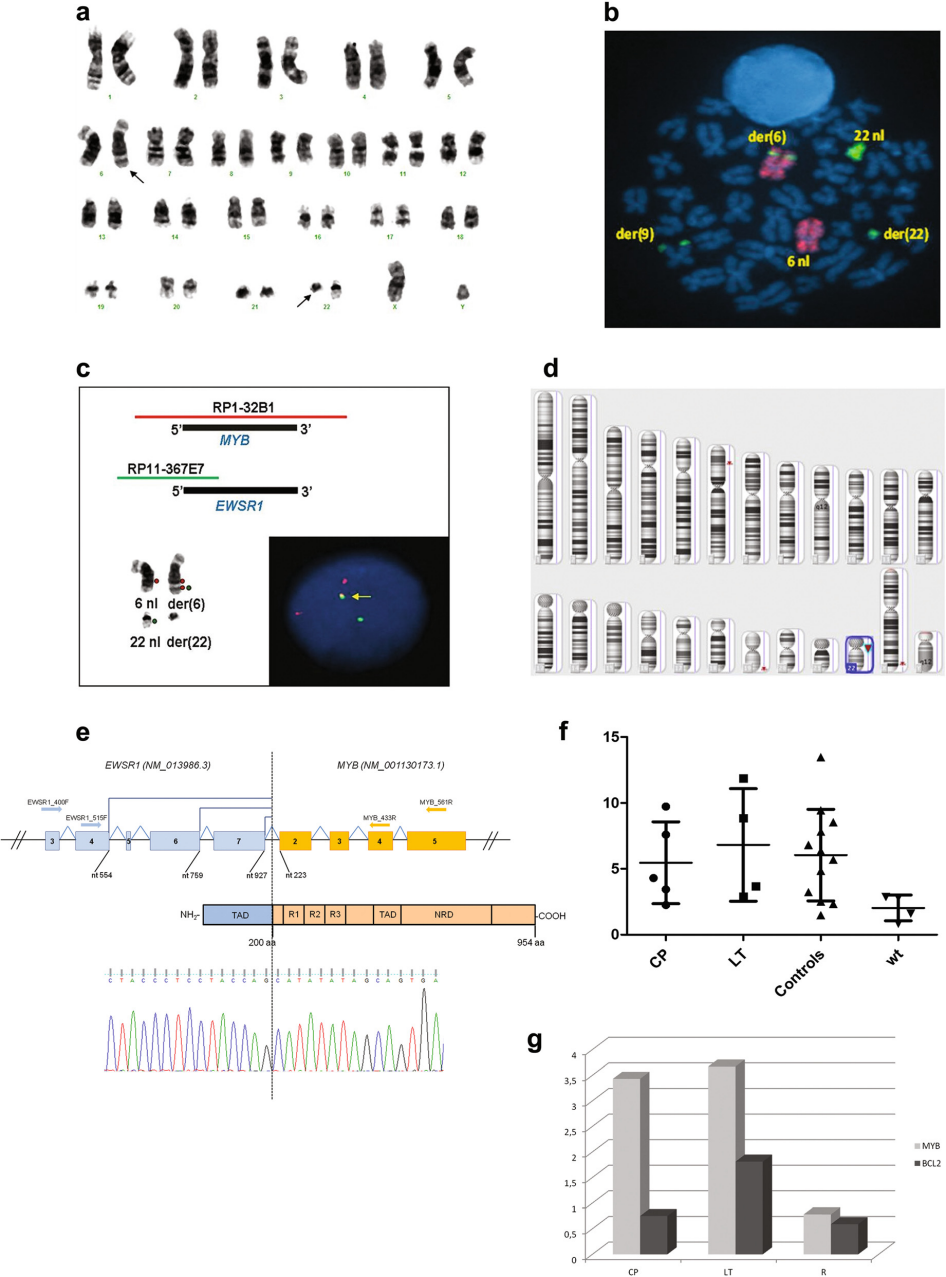
**a**) G-banded karyotype bone marrow cells at leukemic transformation showed ins(6;22)(q23.3;q11) and der(22q) (*black arrows*). **b**) Metaphase FISH experiment with Whole Chromosome Paint (WCP) 6 (*Texas Red*) and WCP 22 (FITC). Genomic material of chromosome 22 was present on der(6) and der(9) long arms. **c**) Schema of clones RP1-32B1 (*MYB*) (*Spectrum Orange*) and RP11-367E7 (*EWSR1*) (*Spectrum Green*) and their mapping. Normal and derivative chromosomes 6 and 22 hybridization patterns; a green/orange fusion signal was present on der(6). A fusion signal was found in interphase nuclei (*yellow arrow*). **d**) SNPa analysis detected loss at 22q (*red triangle*) in BM sample at leukemic transformation. **e**) Schema of the *EWSR1*-*MYB* fusion gene and putative protein. **f**) *MYB* expression analysis in four combined cases at chronic phase and leukemic transformation and in a positive control group (4 *RUNX1*-*RUNX1T1* AML, 4 *MLL*-positive AML, and 4 *BCR*-*ABL1*-positive B-cell ALL). **g**) Longitudinal expression of *MYB* and *BCL2* in the index case. Data are reported for one representative of three independent experiments. Abbreviations: der, derivative chromosome; nl, normal; TAD, transcriptional-activating domain; R1, R2, R3, imperfect aminoacidic repeats; NRD, negative regulatory domain; CP, chronic phase; LT, leukemic transformation; wt, wild-type; R, relapse

In January 2011, a 64-year-old man was referred to our Department with leukocytosis (WBC 20.100/uL with 71.8 % neutrophils), macrocytic anemia (Hb 11,5 g/dL; MCV 104 fl), splenomegaly, and hepatomegaly. Bone marrow (BM) biopsy revealed PMF with grade 1 fibrosis; karyotype was normal. The *JAK2*^V617F^ mutation was found at 60 % allelic burden. After 4 months the patient developed an acute myeloid leukemia (AML; M2 morphology) and acquired an ins(6;22)(q23.3;q11) in the 20 metaphases analyzed (Fig. 1a). He was treated according to the FLAI protocol [13] and obtained hematologic and cytogenetic remission. Myeloproliferative features, including *JAK2*^V617F^, persisted. Demethylating treatment with 5-azacytidine (5-AZA) was administered for 8 cycles, reducing anemia and splenomegaly, but treatment was discontinued because the patient developed a gastric adenocarcinoma. He died 30 months after PMF was diagnosed.

## Methods

### Molecular-cytogenetic and mutational analyses

All molecular and cytogenetic studies were carried out on BM samples. Multi-FISH (24XCyte Multi-colour probe kit, MetaSystems), whole chromosome paints for chromosomes 6, 9 and 22 (Cytocell, Milan, Italy), and metaphase FISH with DNA clones for *MYB* (G248P8686G9 and G248P89100B2), *EWSR1* (G248P89991F7 and G248P80286D12), 9q33.1-q33.3, and 22q11-q12 regions, were done at leukemic transformation (Additional file 1 and Additional file 2: Tables S1, S2, S3). A *EWSR1-MYB* FISH assay (RP1-32B1 for *MYB* and RP11-367E7 for *EWSR1*) was performed at leukemic transformation, diagnosis and post-chemotherapy. The *EWSR1-MYB* assay was also applied to a cohort of 7 PMF and 3 leukemia groups (Additional file 1). Single Nucleotide Polymorphism array (SNPa) experiments were performed at initial PMF and at leukemic transformation; targeted mutational analysis of *DNMT3A*, *SETBP1*, *EZH2*, *IDH1*, *IDH2*, *SRSF2*, *ASXL1*, *NRAS*, *TET2* and *TERT* promoter was done at diagnosis, leukemic transformation, and after chemotherapy by DHPLC and Sanger’s Sequencing. For details of experiments see Additional file 1 and Additional file 2: Table S4.

### Reverse transcription-polymerase chain reaction (RT-PCR) and cloning of *EWSR1*-*MYB*

cDNA from each patient sample was amplified using primers *EWSR1*_400F (5′-GCCCACTCAAGGATATGCAC-3′) and *MYB*_561R (5′-TGCTTGGCAATAACAGACCA-3′), for the first amplification round; nested PCR was set up with primers *EWSR1*_515F (5′-CAGACCGCCTATGCAACTTC-3′) and *MYB*_433R (5′-GCACTGCACATCTGTTCGAT-3′). PCR products were cloned and sequenced (Additional file 1).

### Quantitative reverse transcription PCR (qRT-PCR) for *MYB* and *BCL2*

*MYB* expression was investigated in our patient, at all time points, and in 7 PMF cases (four at leukemic transformation and three in paired chronic phase/leukemic transformation) (TaqMan assay probe Hs00920556_m1; Applied Biosystems, Foster City, CA). Negative controls were four healthy BM samples. Positive controls were 12 acute leukemias with high *MYB* expression (4 *RUNX1*-*RUNX1T1* AML, 4 AML with *MLL* translocations, and 4 *BCR*-*ABL1*-positive B-cell ALL) [14]. Expression of *BCL2*, a known MYB target, was also investigated in our case (TaqMan assay probe Hs00608023_m1, Applied Biosystems). Amplifications were normalized to the endogenous reference control *ABL1* (Hs00245445_m1, Applied Biosystems) (Additional file 1).

## Results

### Molecular cytogenetic and mutational analyses

Multi-FISH and whole chromosome paints showed chromosome 22q11-q12 material inserted into chromosome 6q23 and revealed an additional rearrangement between der(22) and an apparently normal chromosome 9 (Fig. 1b) (Additional file 3: Figure S1). The breakpoint occurred at 6q23 within the *MYB* oncogene (Additional file 3: Figure S2). Fosmids for *EWSR1* and the *EWSR1-MYB* assay showed that the 5′*EWSR1* was inserted into the *MYB* locus (Fig.1c) (Additional file 3: Figure S2). The *EWSR1-MYB* rearrangement was not detected at chronic phase or in 3 consecutive samples analyzed after chemotherapy and during treatment with 5-AZA (Table 1).

**Table 1:** Longitudinal cytogenetic and molecular studies in our patient with rapidly evolving PMF

	PMF diagnosis	Leukemic transformation	Post-consolidation therapy	*Monitoring +3 months	*Monitoring +14 months
Karyotype	46,XY [20]	46,XY,ins(6;22)(q23q11q12),del(22)(q11) [20]	46,XY [20]	46,XY [20]	46,XY [20]
*JAK2* ^V617F^ allele burden	60 %	63 %	70 %	n.d.	36 %
*TET2* c.2732_2733insC, p.A912Cfs*12; .c.3781C>T, p.R1261C	yes	yes	yes	n.d.	n.d.
*SRSF2 *c.284C>A p.P95H	yes	yes	yes	n.d.	n.d.
SNPa	cnLOH 12q11-12q24.33	cnLOH: 12q11-12q24.33	n.d.	n.d.	n.d.
		LOSS: 22q11.1			
I-FISH: *EWSR1-MYB*	negative	positive	negative	negative	negative
RT-PCR: *EWSR1-MYB*	negative	positive	negative	n.d.	n.d.

n.d. not done

*from leukemia transformation

SNPa detected a 96 Mb copy neutral loss of heterozygosity (cnLOH) at 12q11-12q24.33 at chronic phase and leukemic transformation. Lack of germinal material precluded definition as a congenital or acquired event. Applying a 50 Kb filter revealed a 99 kb loss at 22q11.1 (cytostart 17585764 - cytoend 17684472) only at leukemic transformation (Fig. 1d) (Additional file 3: Figure S3). *SRSF2* (c.284C > A; p.P95H) and *TET2* (c.3781C > T; p.R1261C) (c.2732_2733insC; p.A912Cfs*12) mutations were found, and confirmed, at all disease stages (Table 1).

### RT-PCR and qRT-PCR

At leukemic transformation an in-frame fusion transcript *EWSR1-MYB* with breakpoint in *EWSR1* exon 7 (nt 927) (NM_013986.3) and *MYB* exon 2 (nt 223) (NM_001130173.1) was detected. The 954 aminoacids predicted fusion protein retained the EWSR1 transcriptional-activating domain (TAD) at the N-terminal and all MYB functional domains at the C-terminal (Fig. 1e). After chemotherapy, at hematological and cytogenetic remission, the *EWSR1-MYB* fusion disappeared. At leukemic phase, *MYB* and *BCL2* expression was respectively ≥4.7 and ≥2.8 fold higher than at remission. High *MYB* expression was detected in 7 PMF and in the 3 groups of acute leukemia used as positive controls (Fig. 1f).

## Discussion

This case of *JAK2*-positive PMF with very rapid evolution to AML had a specific mutational profile at diagnosis bearing mutations of *JAK2, SRSF2* and *TET2.* A complex rearrangement involving chromosomes 6, 9, and 22, which produced a previously undescribed *EWSR1-MYB* fusion, underlies the leukemic transformation.

EWSR1 is a member of the TET (TLS, EWSR1, TAFII68) protein family which acts as multifunctional protein and regulates transcription and mRNA splicing to maintain cellular homeostasis. Although well known in soft tissue sarcoma, *EWSR1* fusions have only occasionally been detected in leukemia [15–19]. Interestingly, all EWSR1 fusions retained the TAD portion at the N-terminal and the partner DNA Binding Domain (DBD) at the C-terminal. Since EWSR1-FLI1, the most frequent sarcoma-associated fusion, acted as an aberrant transcription factor, deregulating several targets [20], one might speculate that the EWSR1-MYB fusion in our patient also acted as an aberrant transcription factor, altering the *MYB* transcriptional program.

MYB is a leucin zipper transcription factor which plays a key role in cell proliferation and differentiation [21–23]. High level of *MYB* is common in AML associated with *BCR-ABL1, RUNX1-RUNX1T1, MYST3-CREBBP*, or *MLL-* rearrangements [11, 12, 24]. Chromosomal aberrations placing *MYB* under the promoter of a partner gene, such as *TCRB-MYB* in T-cell ALL [14, 25] or in close proximity of super-enhancer regions, such as *MYB-NFIB* in adenoid cystic carcinoma [26], caused *MYB* over-expression. It is worth noting, however, that besides promoting tumour growth through *MYB* over-expression, the *MYB* fusions might have their own oncogenic potential as demonstrated for the *MYB-QKI* in pediatric angiocentric glioma, which drives tumor development in vitro and in vivo models [27].

High *MYB* level confers self-renewal properties on leukemic cells and blocks differentiation, thus it has been regarded as a putative therapeutic target [11, 12]. Indeed, in vitro and in vivo studies showed that a slight *MYB* drop is enough to block leukemic cell proliferation without affecting normal haematopoiesis [28]. The present study confirmed two previous reports of high *MYB* expression in PMF [29, 30] as it was detected in all cases at chronic phase with a rising trend at leukemic transformation (Fig. 1f).

In determining whether and how the aberrant EWSR1-MYB transcription factor played a role in rapid disease progression, paired longitudinal studies showed that the *EWSR1-MYB* fusion was closely linked to leukemic transformation, as it was not detected at diagnosis and disappeared after treatment. As a functional consequence of *MYB* deregulation we investigated *BCL2* expression as it is a well-known MYB target which acts as an anti-apoptotic onco-suppressor. Recent findings in leukemia xenograft models showed that sustained *MYB* expression maintained high *BCL2* expression and consequently, inhibited leukemic cell death [10]. In line with these data, we found that *MYB* as well as *BCL2* expression was higher at leukemic transformation than after treatment (Fig. 1g).

Our patient responded to treatment by regressing from AML to PMF maintaining *JAK2*, *TET2* and *SRSF2* mutations. Therefore, the *JAK2*-positive clone, showed a constant allele burden in all disease phases, marking the PMF stem line which persisted throughout disease, while the *EWSR1-MYB* fusion was the hallmark of the leukemic clone. Whether *EWSR1-MYB* affected a *JAK2*^V617F^ cell or developed as an independent clone, could not be established. Interestingly Engle E.K. et al. [31], identified *MYB* mutations in a complex PMF subclonal branching.

## Conclusion

We report a unique case of *JAK2*^V617F^ PMF. Rapid leukemia transformation, due to complex cytogenetic rearrangement, produced a previously undescribed EWSR1-MYB fusion that appeared to act as an aberrant transcription factor deregulating *BCL2*. Our study provided new insights to point to MYB as a good molecular target in patients presenting with high-risk PMF [11, 32].

## Additional files

Additional file 1: Detailed materials/methods and results. (DOC 68 kb) Additional file 2: Table S1. FISH clones for chromosome 6 long arm breakpoint characterization. **Table S2.** FISH clones for chromosome 22 long arm telomeric breakpoint characterization. **Table S3.** FISH clones for chromosome 9 long arm breakpoint characterization. **Table S4.** Genes, amplicons and primers for mutational analysis. (DOC 991 kb) Additional file 3: Figure S1. Multi-FISH analysis showed a complex rearrangement between chromosomes 6, 9 and 22 (yellow arrows). **Figure S2.** A) Chromosome 6q23 breakpoint within *MYB*. B) Chromosome 22q11.2 breakpoint within *EWSR1*. **Figure S3.** A) SNPa findings at PMF diagnosis: no CNV with 100 and 50 Kb filters (left panel) but LOH at 12q (right panel, black arrow). B) SNPa findings at leukemic transformation: a genomic loss, at 22q11.2, was identified with a 50 Kb filter (left panel, red arrow); LOH at 12q (right panel, red arrow). (DOC 3447 kb)

## Abbreviations

AML: Acute myeloid leukemia
BM: Bone marrow
DBD: DNA binding domain
DHPLC: Denaturing high performance liquid chromatography
ET: Essential thrombocytopenia
FISH: Fluorescence in situ hybridization
Hb: Haemoglobin
I-FISH: Interphase-FISH
MCV: Mean corpuscular volume
Ph-MPN: Philadelphia negative myeloproliferative neoplasia
PMF: Primary myelofibrosis
PV: Polycythemia vera
qRT-PCR: Quantitative reverse transcription-polymerase chain reaction
RT-PCR: Reverse transcription-polymerase chain reaction
SNPa: Single nucleotide polymorphism array
TAD: Transcriptional-activating domain
WBC: White blood cell
