# Supplementary material for: MYB deregulation from a EWSR1-MYB fusion at leukemic evolution of a JAK2V617F positive primary myelofibrosis
Source: Mol Cytogenet. 2016 Sep 1;9(1):68. doi: 10.1186/s13039-016-0277-1 (PMC5009546; doi:10.1186/s13039-016-0277-1)
Supplement: Additional file 1: — Detailed materials/methods and results. (DOC 68 kb) [file 13039_2016_277_MOESM1_ESM.doc]

**Additional file 1**

***MYB* deregulation from a *EWSR1*-*MYB* fusion at leukemic evolution of a *JAK2*^V617F^ positive primary myelofibrosis**

**Tiziana Pierini^1*^, Danika Di Giacomo^1*^, Valentina Pierini^1^, Paolo Gorello^1^, Gianluca Barba^1^, Anair Graciela Lema Fernandez^1^, Fabrizia Pellanera^1^, Tamara Iannotti^1^, Franca Falzetti^1^, Roberta La Starza^1^, Cristina Mecucci1^**

***co-authorship**

**Institutional address**

^1^Hematology and Bone Marrow Transplantation Unit, University of Perugia, C.R.E.O., Perugia, Italy

**^Correspondence:** Cristina Mecucci MD PhD, Hematology Unit, C.R.E.O. piazzale Menghini n.9, 06132 Perugia, Italy; Phone 075 5783808, fax 075 5783691, e-mail: [cristina.mecucci@unipg.it](mailto:cristina.mecucci@unipg.it)

**Material and methods**

**Molecular-cytogenetic and mutational analyses**

FISH was performed in all patient’s samples and in controls: 7 PMF cases (4 at leukemic transformation and 3 in paired chronic phase/leukemic transformation samples) and 3 acute leukemia groups (4 *RUNX1*-*RUNX1T1* AML, 4 *MLL*-positive AML, and 4 *BCR*-*ABL1* positive B-cell ALL). Analysis was carried out on 200 nuclei/cells per experiment with fluorescence microscopy using an Olympus BX61 (Olympus, Milan, Italy) equipped with a highly sensitive camera JAI (Copenhagen, Denmark) and CytoVision 4.5.4 software (Genetix, New Milton, Hampshire, UK). All homegrown FISH probes were selected by NCBI Map Viewer, UCSC Genome Browser and Database of Genomic Variants molecular databases and are reported in Additional file 2: Tables S1, S2, S3 [1-3]. All PMF were tested with the LSI BCR-ABL1 ES dual color translocation probe (Vysis, Abbott Molecular). Multi-FISH was analyzed with a fluorescence microscope Axio Imager.Z2 (Carl Zeiss Microscopy, Germany) equipped with camera Cool Cube1 and "Isis" software (Metasystems) on 5 abnormal metaphases obtained from bone marrow (BM) samples at leukemia evolution.

Copy number alterations (CNAs) and copy-number-neutral loss of heterozygosity (cnLOH) were investigated, at PMF diagnosis and at leukemic transformation, with CytoScan HD human Affymetrix SNPa platform (Affymetrix Santa Clara, CA) in accordance with the manufacturer’s instructions (CytoScan assay user manual and Cytogenetics assay protocol user manual, Affymetrix). Analysis was performed using Affymetrix GeneChip Command Console (AGCC) software and Affymetrix Chromosome Analysis Suite 2.0 (ChAS) software. For accurate state detection, filters were set at 100 and 50 kb for CNAs and at 10 Mb for cnLOH. Data refer to NetAffx Build 32.3 (hg19) database [4].

Mutational analysis of *DNMT3A* exons 15, 19-23 [NM_175629.2]; *ASXL1* exon 13 [NM_015338.5]; *NRAS* exons 2, 3 [NM_002524.4]; *SETBP1* exon 4 [NM_015559.2]; *EZH2* exons 3-21 [NM_004456.4]; *IDH1* exon 4 [NM_005896.3]; *IDH2* exon 4 [NM_002168.3]; *SRSF2* exons 1, 2 [NM_003016.4], *TET2* exons 3-11 [NM_001127208.1] and *TERT* promoter was done by PCR-based Denaturing High Performance Liquid Chromatography (DHPLC) WAVE^TM^ 4500 HT SYSTEM (Transgenomic) and Sanger’s Sequencing, at diagnosis, leukemic transformation and after treatment (Additional file 2: Tables S4).

**Reverse transcription-polymerase chain reaction (RT-PCR) and cloning of *EWSR1*-*MYB*.**

Experiments were executed on biological samples from the patient. Total RNA was extracted by Trizol (Invitrogen, Carlsbad, CA, USA) from bone marrow cells and 1μg was reverse transcribed using Thermoscript (Invitrogen) according to the manufacturer’s protocol. PCR products were sub-cloned into pCR 2.1 TOPO vector (Invitrogen), sequenced with ABI 3500 (Applied Biosystems) and analyzed using the BLAST program (NCBI, http://blast.ncbi.nlm.nih.gov/Blast.cgi) and BLAT Genome Search ([*http://genome.ucsc.edu/cgi-bin/hgBlat*](http://genome.ucsc.edu/cgi-bin/hgBlat)) programs.

**Quantitative reverse transcription PCR (qRT-PCR) for *MYB* and *BCL2***

*MYB* expression was analyzed on biological samples from our patient and in 7 PMF cases (4 at leukemic transformation and 3 in paired chronic phase/leukemic transformation). Negative controls were 4 healthy BM samples while positive controls for *MYB* over-expression were 12 leukemia cases (4 *RUNX1*-*RUNX1T1* AML, 4 *MLL*-positive AML, and 4 *BCR*-*ABL1* positive B-cell ALL). 1µg of total RNA was retrotranscribed using 100U of SuperscriptII (Invitrogen) and esa-random primers (Invitrogen), following the manufacturer’s instructions. All samples were tested in triplicate using Light Cycler 480 (LC480, Roche). Fluorescence data were analyzed with the software version 1.5 and Second Derivative Maximum method; gene expression was expressed as Cp (Crossing point) values.

**Results**

**Molecular cytogenetic and mutational analyses**

FISH showed that chromosomes 6, 9 and 22 were involved in a complex rearrangement (Fig. 1 and Additional file 3: Figure S1). At chromosome 9, the breakpoint was narrowed to a 180 Kb region between RP11-356B19 and RP11-228B15 (Additional file 2: Table S3). At 6q23 band splitting of G248P8686G9 (green) and G248P89100B2 (orange), which were both retained in der(6), indicated the breakpoint fell within *MYB* (Additional file 3: Figure S2). Splitting of LSI EWSR1 probe (Vysis-Abbott) and of fosmid G248P89991F7 (green) showed that the telomeric 22q breakpoint fell within the *EWSR1* gene. The 5’*EWSR1* was inserted into the 6q23 band while the 3’*EWSR1* translocated to an apparently normal chromosome 9 long arm (Additional file 3: Figure S2). A double colour FISH with RP11-367E7 and RP1-32B1 detected a *EWSR1*-*MYB* fusion (Fig. 1) at leukemic evolution but not at PMF diagnosis or after treatment. The 22q centromeric breakpoint was narrowed to a 150 Kb region flanked by RP11-701M12 (deleted) and fosmid G248P8698F7 (translocated to the 6q23 and fused with fosmid G248P8686G9) (Additional file 2: Table S2). The *BCR-ABL1* FISH assay was negative in all PMF cases.

SNPa analysis at PMF diagnosis did not show any CNAs. Applying a 50 Kb filter a 99 kb loss was detected at 22q11.1 (cytostart 17585764-cytoend 17684472) at leukemic transformation. A 96 Mb cnLOH was detected at 12q11-12q24.33, but its germline or acquired nature could not be established (Additional file 3: Figure S3). *SRSF2* (c.284 C>A; p.P95H) and *TET2* (c.3781 C>T; p.R1261C) (c.2732_2733insC; p.A912Cfs*12) mutations were detected at all time points.

**REFERENCES**

1. http://www.ncbi.nim.nih.gov/
2. http://genome.ucsc.edu/
3. http://dgv.tcag.ca/dgv/app/home
4. La Starza R, Borga C, Barba G, et al. Genetic profile of T-cell acute lymphoblastic leukemias with MYC translocations. Blood. 2014;124(24):3577-82.
