# Supplementary material for: MYB deregulation from a EWSR1-MYB fusion at leukemic evolution of a JAK2V617F positive primary myelofibrosis
Source: Mol Cytogenet. 2016 Sep 1;9(1):68. doi: 10.1186/s13039-016-0277-1 (PMC5009546; doi:10.1186/s13039-016-0277-1)
Supplement: Additional file 2: Table S1. — FISH clones for chromosome 6 long arm breakpoint characterization. Table S2. FISH clones for chromosome 22 long arm telomeric breakpoint characterization. Table S3. FISH clones for chromosome 9 long arm breakpoint characterization. Table S4. Genes, amplicons and primers for mutational analysis. (DOC 991 kb) [file 13039_2016_277_MOESM2_ESM.doc]

**Additional file 2: additional Tables**

***MYB* deregulation from a *EWSR1*-*MYB* fusion at leukemic evolution of a *JAK2*^V617F^ positive primary myelofibrosis**

**Tiziana Pierini^1*^, Danika Di Giacomo^1*^, Valentina Pierini^1^, Paolo Gorello^1^, Gianluca Barba^1^, Anair Graciela Lema Fernandez^1^, Fabrizia Pellanera^1^, Tamara Iannotti^1^, Franca Falzetti^1^, Roberta La Starza^1^, Cristina Mecucci1^**

***co-authorship**

**Institutional address**

^1^Hematology and Bone Marrow Transplantation Unit, University of Perugia, C.R.E.O., Perugia, Italy

**^Correspondence:** Cristina Mecucci MD PhD, Hematology Unit, C.R.E.O. piazzale Menghini n.9, 06132 Perugia, Italy; Phone 075 5783808, fax 075 5783691, e-mail: [cristina.mecucci@unipg.it](mailto:cristina.mecucci@unipg.it)


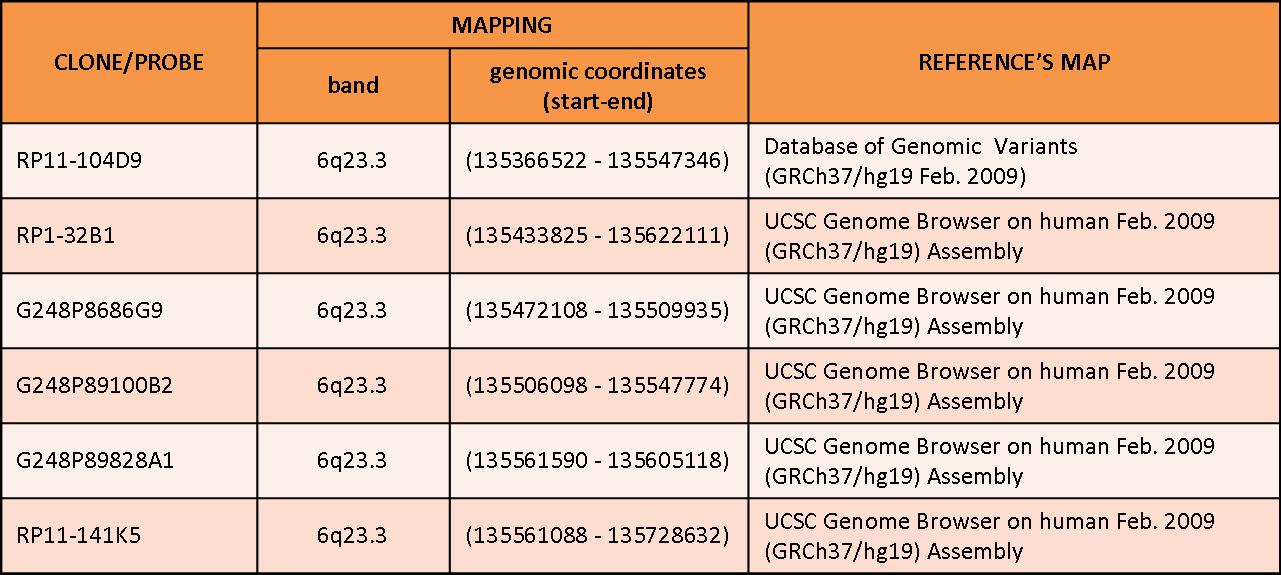
**Table S1.** FISH clones for chromosome 6 long arm breakpoint characterization.

**Table S2.** FISH clones for chromosome 22 long arm telomeric breakpoint characterization.

**
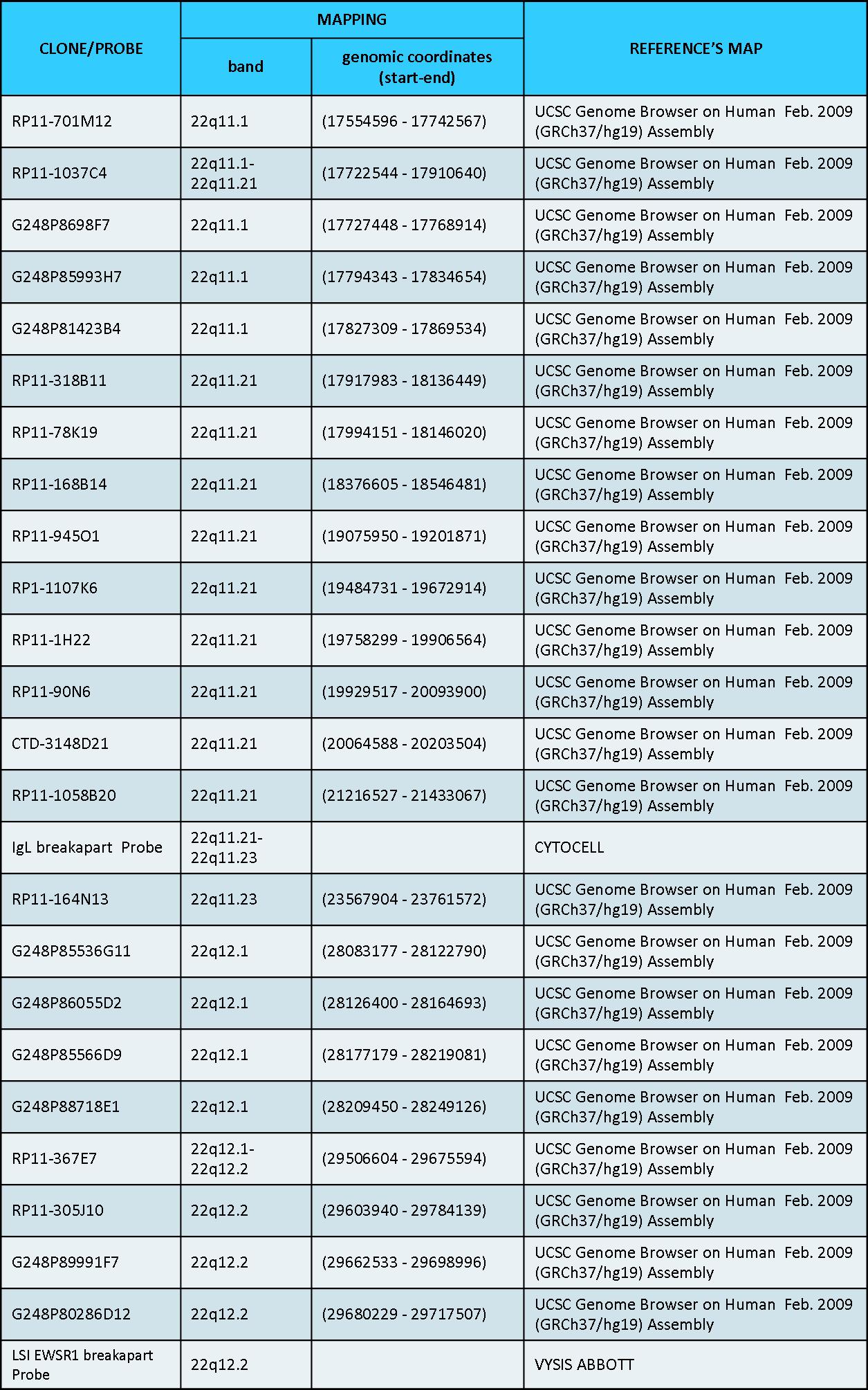
**

**Table S3.** FISH clones for chromosome 9 long arm breakpoint characterization.

**
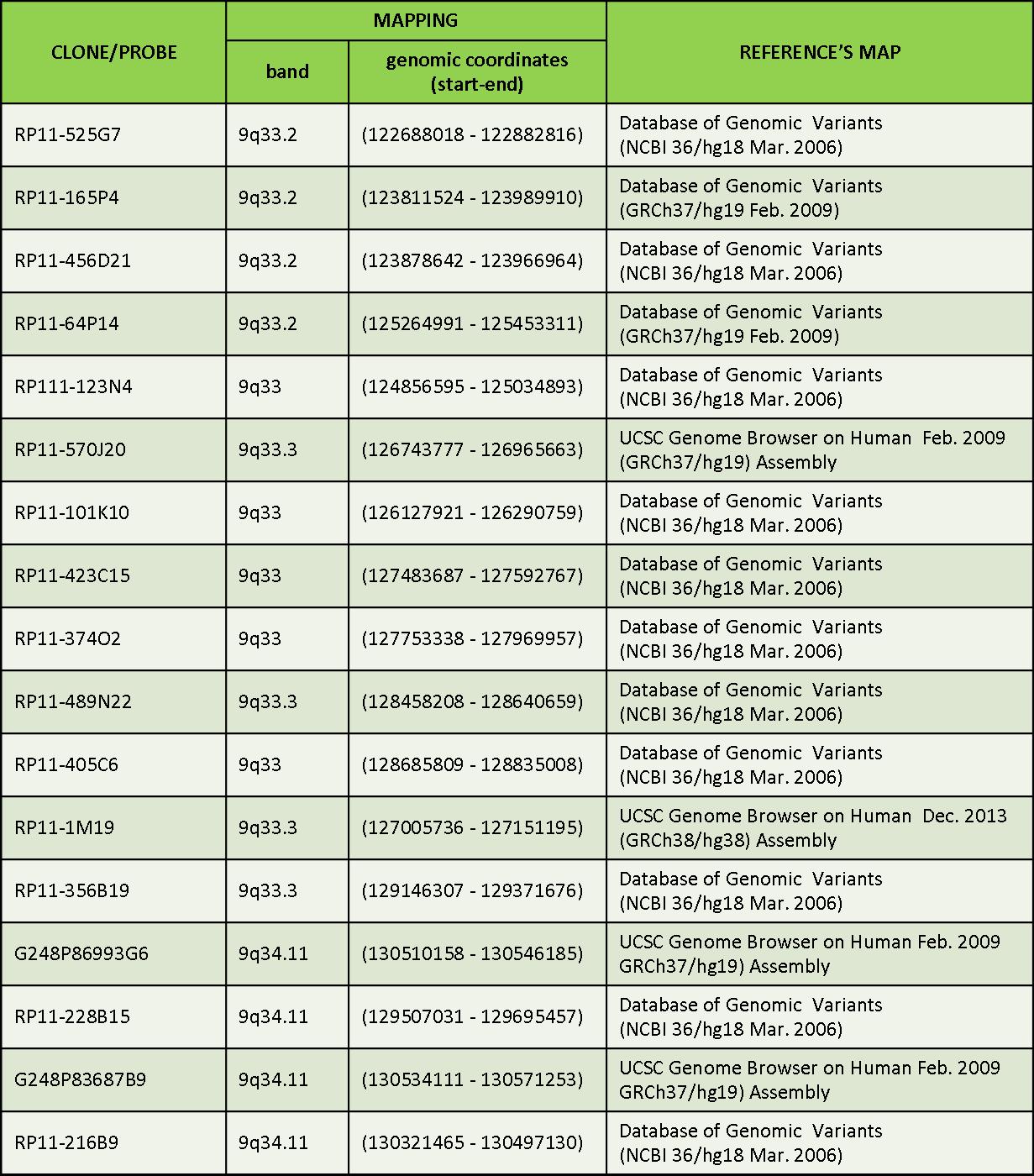
**

**Table S4:** Genes, amplicons and primers for mutational analysis.
