# Supplementary material for: MYB deregulation from a EWSR1-MYB fusion at leukemic evolution of a JAK2V617F positive primary myelofibrosis
Source: Mol Cytogenet. 2016 Sep 1;9(1):68. doi: 10.1186/s13039-016-0277-1 (PMC5009546; doi:10.1186/s13039-016-0277-1)
Supplement: Additional file 3: Figure S1. — Multi-FISH analysis showed a complex rearrangement between chromosomes 6, 9 and 22 (yellow arrows). Figure S2. A) Chromosome 6q23 breakpoint within MYB. B) Chromosome 22q11.2 breakpoint within EWSR1. Figure S3. A) SNPa findings at PMF diagnosis: no CNV with 100 and 50 Kb filters (left panel) but LOH at 12q (right panel, black arrow). B) SNPa findings at leukemic transformation: a genomic loss, at 22q11.2, was identified with a 50 Kb filter (left panel, red arrow); LOH at 12q (right panel, red arrow). (DOC 3447 kb) [file 13039_2016_277_MOESM3_ESM.doc]

**Additional file 3: additional Figures**

***MYB* deregulation from a *EWSR1*-*MYB* fusion at leukemic evolution of a *JAK2*^V617F^ positive primary myelofibrosis**

**Tiziana Pierini^1*^, Danika Di Giacomo^1*^, Valentina Pierini^1^, Paolo Gorello^1^, Gianluca Barba^1^, Anair Graciela Lema Fernandez^1^, Fabrizia Pellanera^1^, Tamara Iannotti^1^, Franca Falzetti^1^, Roberta La Starza^1^, Cristina Mecucci1^**

***co-authorship**

**Institutional address**

^1^Hematology and Bone Marrow Transplantation Unit, University of Perugia, C.R.E.O., Perugia, Italy

**^Correspondence:** Cristina Mecucci MD PhD, Hematology Unit, C.R.E.O. piazzale Menghini n.9, 06132 Perugia, Italy; Phone 075 5783808, fax 075 5783691, e-mail: [cristina.mecucci@unipg.it](mailto:cristina.mecucci@unipg.it)

**Figure S1**: Multi-FISH analysis showed a complex rearrangement between chromosomes 6, 9 and 22 (yellow arrows).


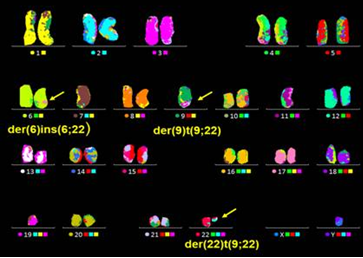


**Figure S2.** A) Chromosome 6q23 breakpoint within *MYB*. B) Chromosome 22q11.2 breakpoint within *EWSR1*.

**Figure S3.** A) SNPa findings at PMF diagnosis: no CNV with 100 and 50 Kb filters (left panel) but LOH at 12q (right panel, black arrow). B) SNPa findings at leukemic transformation: a genomic loss, at 22q11.2, was identified with a 50 Kb filter (left panel, red arrow); LOH at 12q (right panel, red arrow).


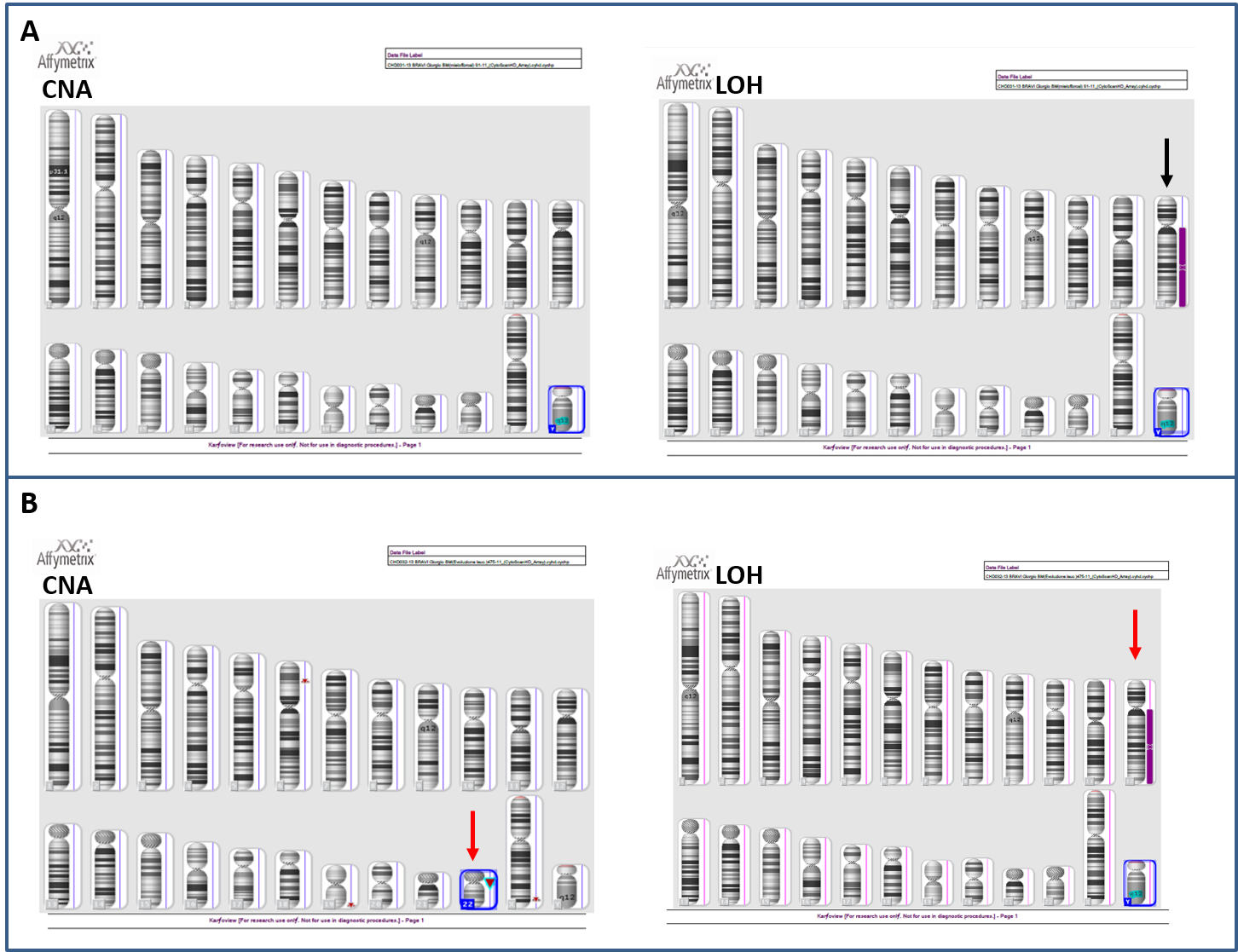


**Abbreviations:** **multi-FISH**, multi-fluorescent in situ hybridization; M-FISH, metaphase-fluorescent in situ hybridization; **der**, derivative chromosome; **ins**, insertion; **t**, translocation; **nl**, normal; **SNPa**, single nucleotide polymorphism array; **CNV**, copy number variation; **CNA**, copy number alteration; **LOH**, loss of heterozygosity**; PMF**, primary myelofibrosis;.
